# The blood fluke *Schistosoma mansoni* cleaves the coagulation protein high molecular weight kininogen (HK) but does not generate the vasodilator bradykinin

**DOI:** 10.1186/s13071-018-2704-0

**Published:** 2018-03-14

**Authors:** Qiang Wang, Akram A. Da’dara, Patrick J. Skelly

**Affiliations:** Molecular Helminthology Laboratory, Department of Infectious Disease and Global Health, Cummings School of Veterinary Medicine, Tufts University, North Grafton, MA USA

**Keywords:** *Schistosoma mansoni*, Coagulation, High molecular weight kininogen, Bradykinin, Calpain

## Abstract

**Background:**

Schistosomes are blood dwelling parasitic worms that cause the debilitating disease schistosomiasis. Here we examined the influence of the parasites on their external environment by monitoring the impact of adult *Schistosoma mansoni* worms on the murine plasma proteome in vitro and, in particular, on how the worms affect the blood coagulation protein high molecular weight kininogen (HK).

**Methods:**

Following the incubation of adult schistosomes in murine plasma, two-dimensional differential in-gel electrophoresis (2D-DIGE) was conducted to look for changes in the plasma proteome compared with control plasma. A major change to the blood protein kininogen (HK) was observed, and the interaction of *Schistosoma mansoni* parasite with this protein alone was then investigated by western blot analysis and activity assays. Finally, the generation of bradykinin from HK was monitored using a bradykinin detection kit.

**Results:**

The most striking change to the plasma proteome concerned HK; while the full-length protein was more abundant in control plasma, carboxyl-terminal truncated forms were more abundant in plasma that contained schistosomes. Incubating parasites in buffer with pure HK followed by Western blot analysis confirmed that human HK is degraded by the worms. The resulting digestion pattern differed from that brought about by kallikrein, a host serine protease that normally acts on HK to release the vasodilator bradykinin. We found that live schistosomes, while digesting HK, do not generate bradykinin nor do they cleave a chromogenic kallikrein substrate. Since the cleavage of HK by the worms is not impeded by the serine protease inhibitor PMSF but is blocked by the cysteine protease inhibitor E64c, we hypothesize that schistosome tegumental cysteine proteases are responsible for HK cleavage.

**Conclusions:**

Since proteomic and biochemical studies have revealed that the schistosome tegument contains two cysteine proteases belonging to the calpain family (SmCalp1 and SmCalp2) we conclude that these are likely responsible for the HK cleavage reported here. Schistosome cleavage of HK should help impede blood clotting and inflammation around the worms in vivo and so promote their ease of movement within the vasculature of their hosts.

**Electronic supplementary material:**

The online version of this article (10.1186/s13071-018-2704-0) contains supplementary material, which is available to authorized users.

## Background

Schistosomes are parasitic flatworms that live in the vascular system of vertebrates, causing a chronic, debilitating disease called schistosomiasis. Over 200 million people are estimated to be infected with schistosomes in over 70 countries [1] with another 800 million people at risk of infection [2]. Annual mortality in sub-Saharan Africa alone is over 250,000 people, with many more experiencing chronic morbidity [3, 4]. Three major species infect humans; these are *Schistosoma mansoni*, *S. japonicum* and *S. haematobium* [5]. Schistosomiasis is a water-borne disease, and infection occurs when free-swimming larval parasites called cercariae penetrate the skin. Here, each cercaria transforms into a morphologically and biochemically distinct life stage called the schistosomulum. Schistosomula invade a blood vessel, migrate through the heart and lungs to the liver where the worms mature and males pair with females. *S. mansoni* and *S. japonicum* couples then travel to the mesenteric veins, while *S. haematobium* pairs migrate to the vesicle venous plexus, where egg-laying occurs [6].

We are interested in how schistosomes modify their environment to promote their survival, and we are investigating the molecular capabilities of selected proteins expressed at the worm surface [7–10]. Here, we explored the impact of adult schistosomes on the murine plasma proteome. After incubating schistosomes in plasma, the most dramatic change seen in the plasma proteome is cleavage of high molecular weight kininogen (HK). HK is a circulating blood protein that is involved in the early stages of what is called the intrinsic coagulation pathway. HK is not enzymatically active; it functions as a cofactor in the conversion of coagulation factor XII to its active form (factor XIIa) and the conversion of prekallikrein to its active form (kallikrein) [11]. HK is also necessary for the activation of coagulation factor XI by factor XIIa [11]. In addition to acting as a cofactor, HK can itself be acted upon by kallikrein to generate the 9-amino acid inflammatory mediator bradykinin [12].

It has been reported that schistosome homogenates do possess a kallikrein-like activity, associated with an uncharacterized 66 kDa protein (designated sK1) that can hydrolyze the kallikrein synthetic substrate d-Pro-Phe-Arg-p-nitroanilide [13]. The sK1 protein is localized to the tegumental surface of the adult parasite [13]. Also, an *S. mansoni* cDNA encoding a mouse plasma kallikrein homolog was identified (designated SmSP1) [14]. Since this cloned DNA encodes a ~35 kDa protein, this molecule differs from the larger, sK1 protein. SmSP1 was detected in schistosomula released products and male dorsal spines [14]. These host-exposed localization sites of sK1 and SmSP1 are consistent with schistosomes possessing a kallikrein-like activity.

Here, we monitor the ability of schistosomes to cleave HK ex vivo and compare this to the action of the host HK cleaving enzyme kallikrein. Additionally, we look for the ability of the worms to generate the vasodilator bradykinin. This work is designed to understand better the molecular capabilities of schistosomes that permit them to survive in the vasculature of their hosts for many years.

## Methods

### Parasites and mice

*Schistosoma mansoni* infected *Biomphalaria glabrata* snails (strain NMRI) were obtained from the Schistosomiasis Resource Center at the Biomedical Research Institute (BRI), Rockville MD. Larval schistosomes (cercariae, strain NMRI) were obtained from the infected snails, and schistosomula were prepared as described [15]. Adult male and female parasites were recovered by perfusion from Swiss Webster mice that were infected with 120 *S. mansoni* cercariae seven weeks previously. All parasites were cultured in complete Dulbecco's Modified Eagle Medium/Nutrient Mixture F-12 (DMEM/F12) medium supplemented with 10% heat-inactivated fetal bovine serum, 200 U/ml penicillin and 200 μg/ml streptomycin, 0.2 μM Triiodo-l-thyronine, 1 μM serotonin and 8 μg/ml human insulin and were maintained at 37 °C, in an atmosphere of 5% CO_2_ [16]. All protocols involving animals were approved by the Institutional Animal Care and Use Committees (IACUC) of Tufts University under protocol G2015-113.

### Treatment of mouse plasma with schistosome parasites

To obtain plasma, mouse blood was collected from the tale veins of 10 Swiss Webster mice into a collecting tube containing heparin. Samples were pooled, centrifuged at 13,000× *rpm* for 15 min at 4 °C, and the supernatant (plasma) recovered. Adult parasites were recovered from the culture medium and washed with Roswell Park Memorial Institute (RPMI) medium five times. Approximately 50 pairs of adult worms were incubated in 250 μl of heparinized mouse plasma with 2 mM CaCl_2_ for one hour at 37 °C. As a control, heparinized mouse plasma alone with 2 mM CaCl_2_ was likewise incubated at 37 °C for 1 h. After that, parasites were removed, and plasma samples were submitted to Applied Biomics Inc. (Hayward, CA, USA) for two-dimensional differential in-gel electrophoresis (2D-DIGE) and proteomic analysis.

### Two-dimensional differential in-gel electrophoresis (2D-DIGE)

At Applied Biomics Inc., equal plasma samples were labelled with CyDye fluors: Cy2 (for the control (no parasite) sample) or Cy3 (for the plasma sample incubated with parasites for 1 h). Labeled samples were then mixed with rehydration buffer, and proteins were resolved first by isoelectric focusing and then by sodium dodecyl sulfate-polyacrylamide gel electrophoresis (SDS-PAGE) in the second dimension. The resulting 2-D gel was scanned using a Typhoon image scanner to reveal each of the CyDye signals (Cy2 and Cy3). The scanned image was analyzed using ImageQuant software and comparative analysis of all spots was performed using DeCyder “in-gel” analysis software (Applied Biomics) to generate protein level ratios. To determine the identities of the selected proteins within the gel, spots of interest were picked using an Ettan Spot Picker. Excised spots were subjected to in-gel trypsin digestion followed by mass spectrometry (MS) analysis at Applied Biomics, Inc. Protein identification was based on peptide fingerprint mass mapping (using MS spectra) and peptide fragmentation mapping (using MS/MS spectra). Combined MS and MS/MS spectra were subjected to database searching using GPS Explorer software equipped with the MASCOT search engine to identify proteins from primary murine and schistosome sequence databases.

### High molecular weight kininogen (HK) assay

Schistosomula (~1000) or adult parasites (5) were washed with Hanks balanced salt solution (HBSS) 3 times, and then resuspended in 150 μl assay buffer (20 mM HEPES, 130 mM NaCl, 1 mM EDTA, 10 mM Glucose). An equal volume (150 μl) of 6 mM CaCl_2_, 10 mM 2-mercaptoethanol and 20 ng/μl (180 nM) plasma-purified, human HK (HK-HMW, Molecular Innovations, USA) was then added to each well. Next, parasites were incubated at 37 °C for 1 h. Controls include the same HK solution to which 1 μg of plasma-purified, human kallikrein (Millipore Sigma, Burlington, USA) was added or the HK solution without parasites. In some experiments, the serine protease inhibitor phenylmethylsulfonyl fluoride (PMSF, 0.5 mM, Sigma-Aldrich, St. Louis, USA) or the cysteine protease inhibitor E64c (0.1 mM, E0514, Sigma-Aldrich) was also added to the HK solution containing parasites. At selected time points (routinely, at 6 h and 24 h) 30 μl aliquots were recovered from each treatment group and analyzed by western blotting and for bradykinin generation.

### SDS-PAGE and Western blot analysis

Samples recovered from the HK assay described above were resolved by 4–20% SDS-PAGE (BioRad, Hercules, USA) as previously described [17]. Proteins were then transferred to PVDF membrane and blocked with TBST (Tris-buffered saline, pH 7.5, 0.05% Tween 20) containing 5% dry non-fat milk powder for 1 h at room temperature. The membrane was then incubated with primary anti-HK antibody (Abcam, Cambridge, United Kingdom, 1:1000 dilution) for 1 h at room temperature, followed by washing with TBST buffer for 30 min and incubation with goat anti-rabbit IgG conjugated to horseradish peroxidase (GE Healthcare, Marlborough, USA) at 1:5000, for 1 h at room temperature. The blots were developed using Amersham ECL Detection Reagents (GE Healthcare) according to the manufacturer’s instructions and images were recorded using a ChemiDoc™ Imaging System (Bio-Rad).

### Kallikrein assay

Schistosomula (~1000) or adult parasites (5) were washed with HBSS 3 times, resuspended in 100 μl assay buffer (described above) and placed into a well of a 48-well tissue culture plate. An equal volume (100 μl) of 6 mM CaCl_2_, 10 mM 2-mercaptoethanol, with or without 1 mM PMSF and 800 μM kallikrein substrate (D-Pro-Phe-Arg-pNa, CS-31(02), HYPHEN Biomed, Neuville-sur-Oise, France) was added into each well. The product generated (para-nitro-aniline (pNA)) was monitored at OD 405 nm over time at 37 °C. Control wells contained the kallikrein substrate solution without parasites (negative control) and the kallikrein solution containing plasma purified, human kallikrein (0.02 μg, positive control, Millipore Sigma).

### Bradykinin measurement

Samples recovered at 6 h and 24 h in the HK assay (described above) were analyzed for the presence of the 9-amino acid HK cleavage product bradykinin using a Bradykinin ELISA kit, (Abcam) and following the manufacturer's protocol. In short, bradykinin standards and samples were prepared and added to a series of wells of a capture antibody-coated 96-well plate. Then a biotinylated bradykinin conjugate was added to each well, followed by an anti-bradykinin polyclonal antibody. The mixture was then incubated at room temperature for 2 h. The plate was then washed 5 times using the wash buffer provided, and a streptavidin-HRP solution was added to all wells. Next, the plate was incubated for 30 min, washed a further 5 times before tetramethylbenzidine (TMB) substrate solution was added to all wells. After room temperature incubation for 30 min, stop solution was added and the plate was read at OD 450 nm.

### Statistical analysis

For the kallikrein activity assay, two-way ANOVA was used and *P*-values were considered significant at < 0.05. Statistical analyses were performed using GraphPad Prism 5 (La Jolla, San Diego, CA, USA).

## Results

### Changes in the mouse plasma proteome in the presence of schistosomes

**Fig. 1:**
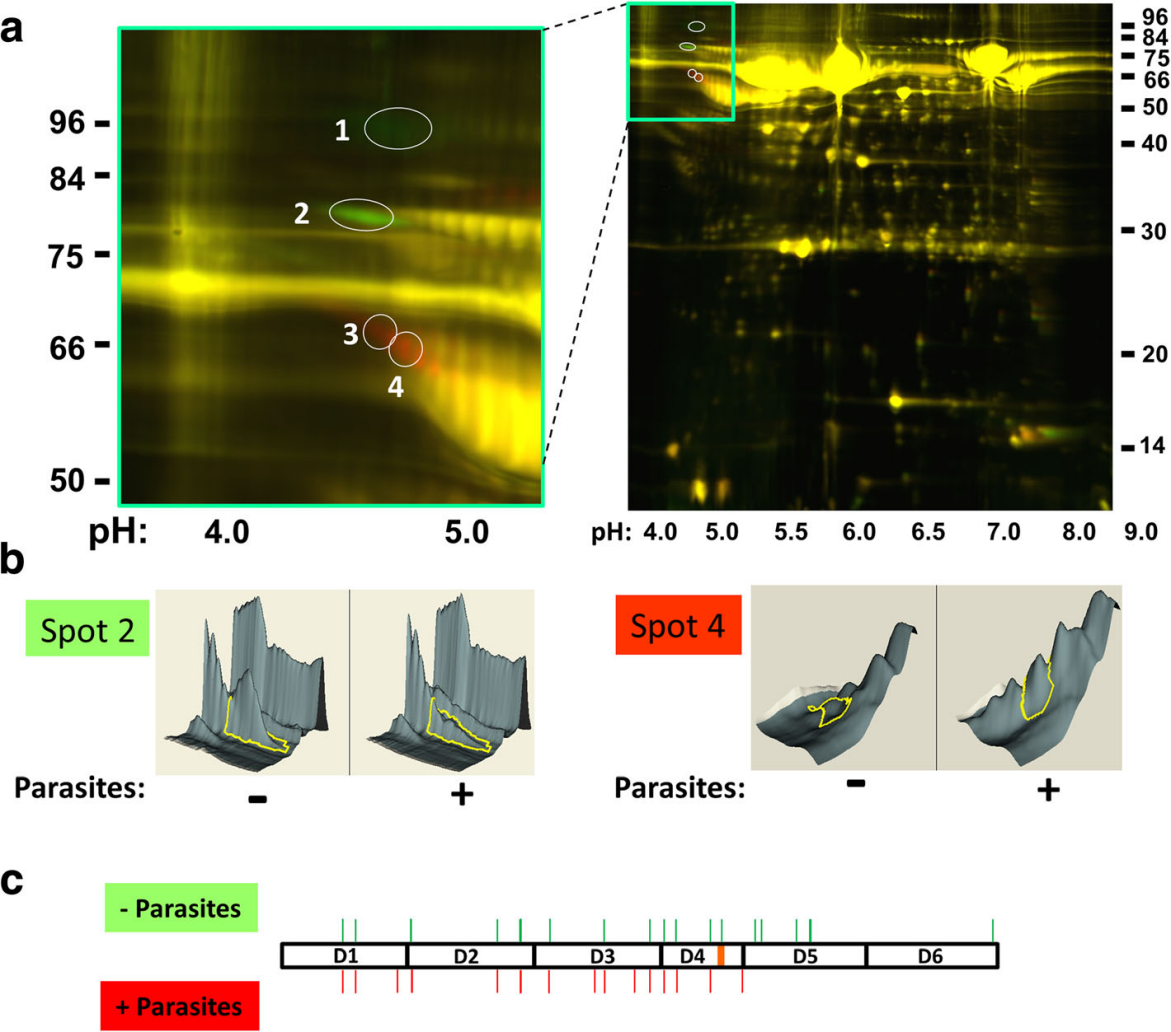
Impact of schistosomes on the mouse plasma proteome. **a** Image (right) showing 2D gel resolution of samples obtained following normal heparinized mouse plasma incubation for 1 h at 37 °C either in the presence of schistosome parasites (red) or without parasites (green). Most proteins are the same in both samples and appear yellow. The numbers on the sides of the images represent molecular mass markers (kDa) and the numbers at the bottom represent pH values. The section containing protein spots of interest is bounded by a green box at the top and this section is magnified and shown on the left. Four spots are circled and numbered. Two exhibits greater relative abundance in the absence of parasites (green spots 1, 2); two are more abundant in the presence of parasites (red spots 3 and 4). **b** Gel spot analysis by DeCyder software. The left panel depicts an analysis of gel spot 2. Here the sizable protein peak (bounded by a yellow line in the figure) that is seen in the control sample (- parasites) is greatly diminished in the sample containing parasites (+ parasites). Spot 4, right panel, shows the opposite effect in which the identified spot is barely detectable in the control sample (-) but becomes prominent in the presence of the worms (+). **c** Diagrammatic representation of the HK protein with its 6 domains (D1-D6). The 9-amino acid vasodilator bradykinin is contained within domain 4 and is indicated by an orange line. The positions of peptides that were identified by mass spectroscopy following trypsin digestion of 2D-DIGE spots 1 and 2 are indicated by thin green lines protruding above the HK protein. Positions of peptides identified by mass spectroscopy following trypsin digestion of 2D-DIGE spots 3 and 4 are indicated by red lines protruding below the protein

As assessed by 2D-DIGE, normal mouse plasma in which adult schistosomes were incubated for 1 h at 37 °C displays a different proteomic profile compared with plasma incubated without schistosomes. The 2D electrophoresis pattern of both plasma samples is shown in Fig. 1a (right) where spots coloured red indicate proteins that are in greater relative abundance in the plasma sample that contained worms while spots coloured green are in greater abundance in the control plasma sample (without worms). The vast majority of proteins display no detectable difference between the two samples and these appear yellow.

In this work, we focus on the most striking differences between the protein profiles of the two plasma samples, highlighted in the boxed area in Fig. 1a. This area is enlarged in the left panel of Fig. 1a and contains four spots (numbered 1–4). Mass spectrometry analysis, following recovery and trypsin digestion of these protein spots, reveals that all are high molecular weight kininogen (HK, also known as kininogen-1; accession designation: KNG1_MOUSE) or fragments thereof. Examination using ImageQuant software reveals that spots 1 and 2 are found in greater relative abundance in the control plasma sample (4.6-fold and 2.1-fold, respectively) and appear green, while red spots 3 and 4 are found in greater relative abundance in the sample that contained worms (3.3-fold and 3.1-fold, respectively). Figure 1b, left panel, depicts an analysis of gel spot 2 using the DeCyder software in which the sizable protein peak (bounded by a yellow line in the figure) that is seen in the control sample (“- parasites”) is greatly diminished in the sample containing parasites (“+ parasites”). DeCyder analysis of spot 4 (Fig. 1b, right panel) shows the opposite effect in that the identified spot is barely detectable in the control sample (left panel) but is strikingly revealed in the presence of the worms (right panel). Figure 1c depicts the HK protein with its 6 domains (D1-D6). The 9-amino acid vasodilator bradykinin is contained within domain 4 (D4) and is depicted in Fig. 1c by an orange line. The positions of peptides that were identified by mass spectroscopy following trypsin digestion of 2D-DIGE spots 1 and 2 are indicated by thin green lines protruding above the HK sequence in Fig. 1c. Peptides identified by mass spectroscopy following trypsin digestion of 2D-DIGE spots 3 and 4 are indicated by red lines protruding below the sequence. Additional file 1: Table S1 provides details regarding the peptides detected. Since no peptides derived from the carboxyl end of HK are detected from protein spots 3 and 4, this suggests that schistosomes are responsible for the generation of carboxyl-truncated forms of HK. The data suggest cleavage of HK in the presence of the parasites beyond HK domain 4. This analysis does not provide clues as to the differences in the migration pattern of HK spot 1 *vs* spot 2 (presumably both are the full-length proteins), nor for differences between spot 3 *vs* spot 4 (truncated forms of HK) although differential post-translational modification (e.g. glycosylation) may contribute. Other differences between the plasma samples, not related to HK, will be explored in a separate publication.

### Analysis of HK cleavage by schistosomes

**Fig. 2:**
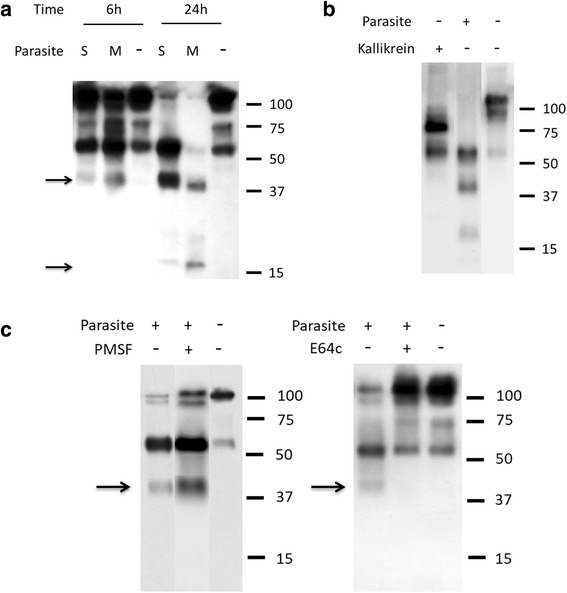
Analysis of HK cleavage by schistosomes using western blotting. **a** High molecular weight kininogen (HK) was incubated in the absence (-) or presence of parasites (S, schistosomula; M, males) for different time periods (6 or 24 h, as indicated). Samples were resolved by SDS-PAGE, blotted to membrane and probed by western blotting. A number of prominent HK degradation products are detected (at ~40 and 16 kDa, arrows) only in the presence of parasites. The numbers on the right indicate molecular mass markers (kDa). **b** Parasites or kallikrein, as indicated, were incubated with HK for 24 h and cleavage products were resolved by SDS-PAGE, blotted to membrane and probed for HK digestion by western blotting. Numbers represent molecular mass markers (kDa, right). **c** HK was incubated for 6 h with parasites either in the presence or absence of serine protease inhibitor PMSF (0.5 mM), as indicated (left panel); or in the presence or absence of cysteine protease inhibitor E64c (0.1mM) (right panel). The presence of PMSF (left panel) does not impede parasite-mediated cleavage of HK. The characteristic ~40 kDa product is indicated by the arrow. In contrast, the presence of E64c (right panel) does impede parasite-mediated cleavage of HK

To validate our proteomic findings regarding HK, we incubated living schistosomes with commercially obtained human HK (purified from plasma) and recovered samples 6 h and 24 h later. These samples were resolved by SDS-PAGE, blotted to a membrane and probed with the polyclonal anti-HK antibody. Results of this analysis are shown in Fig. 2a. At the 6 h time point it is clear that the presence of parasites (either schistosomula (S) or adult male worms (M)) does lead to HK cleavage: a ~40 kDa band is apparent (top arrow, Fig. 2a) that is not seen in the control sample incubated without worms (“-” lane). Prolonged incubation (to 24 h) results in an accumulation of the ~40 kDa band (most apparent in the 24 h schistosomula (S) lane) and the appearance of a second cleavage product at ~16 kDa (bottom arrow, Fig. 2a). Neither is seen in the control sample at 6 h or 24 h. The appearance of these bands is coincident with the diminution in the intensity of the full-length HK protein running at ~120 kDa.

Next, HK was incubated with commercially obtained human kallikrein. Samples recovered 6 h and 24 h after incubation were resolved by SDS-PAGE, blotted to a membrane and probed with the polyclonal anti-HK antibody. The pattern of HK cleavage by kallikrein at the 24 h time point is compared to that generated by schistosomes in Fig. 2b (the 6 h and 24 h samples yielded identical patterns). As expected, kallikrein cleaves HK to generate two major products, a light chain and a heavy chain, as shown in Fig. 2b, left lane. This pattern is very different from that seen following schistosome incubation with HK (Fig. 2b, central lane) where the characteristic 40 kDa and 16 kDa bands are again revealed. A control sample (HK alone) is also shown in Fig. 2b, right lane.

To explore what kind of schistosome protease is involved in substrate cleavage, parasites were incubated with HK in the presence of the serine protease inhibitor PMSF (0.5 mM) or the presence of the cysteine protease inhibitor E64c (0.1 mM) and the results are shown in Fig. 2c. It is clear that adding PMSF does not noticeably impede the ability of schistosomes to cleave HK (Fig. 2c, left panel). The characteristic 40 kDa cleavage fragment (arrow) is seen both in the presence (Fig. 2c, lane +/+) and the absence (lane +/-) of inhibitor. In contrast, adding E64c does block HK cleavage (right panel, lane +/+); the HK profile seen in the presence of E64c is essentially the same as that seen in the control lane (HK alone, lane -/-). Note that following incubation of worms with these inhibitors, no obvious morphological differences were seen between them and control, untreated worms.

### Kallikrein activity assay

**Fig. 3:**
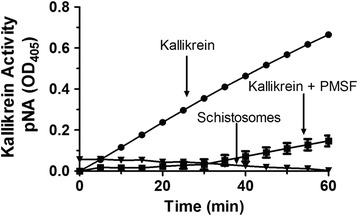
Kallikrein activity assay. Cleavage of kallikrein substrate (H-D-Pro-Phe-Arg-pNa) by living schistosomes (triangle) and human kallikrein (0.02 μg) in the presence (square) or absence (circle) of PMSF (2 mM) (Mean +/- SD, *n* = 3). Released pNa was measured every 5 minutes at OD_405_. All conditions differ significantly from “Kallikrein”; two-way ANOVA: *F*_(24,52)_ = 94.76, *F*_(2,52)_ = 2318.22, *F*_(12,52)_ = 124.82, *P* < 0.0001

The plasma serine protease kallikrein is known to cleave HK to generate the vasodilator bradykinin [18, 19]. Since we have shown here that schistosomes do target HK for cleavage, the question arose as to whether the worms possess kallikrein (or kallikrein-like) activity. To test this, live worms were incubated with a chromogenic kallikrein substrate (D-Pro-Phe-Arg-pNa) [13] whose cleavage was monitored at OD_405_. Figure 3 shows the result of this assay. No convincing cleavage of the product was detected in the presence of the live parasites. Parasites incubated with substrate beyond 1 hour (and tested at 2, 4, 8, 12 and 24 h) similarly failed to generate a detectable product. In contrast, adding human kallikrein to the assay results in clear and rapid cleavage of the substrate (Fig. 3). Adding the serine protease inhibitor PMSF to the assay impedes the ability of kallikrein to cleave this substrate, as expected.

### Bradykinin detection

While the work presented here shows that schistosomes can cleave HK, there is no evidence that they possess kallikrein activity (which would ordinarily release bradykinin from HK). Of course, it remains possible that the worms possess a non-kallikrein activity that nonetheless yields bradykinin. To assess whether the parasites can generate bradykinin as a product of HK cleavage, we employed a competitive bradykinin ELISA assay.

**Fig. 4:**
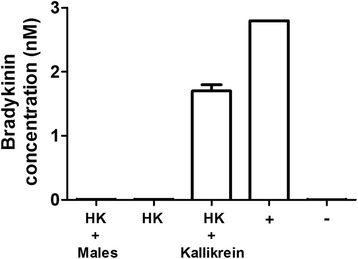
Bradykinin detection. Bradykinin generated following incubation of HK with either male schistosomes (HK + males) or with pure human kallikrein (HK + Kallikrein) or alone (HK) for 24 h at 37^o^C. Positive (+) and negative (-) controls used in this ELISA assay are bradykinin, 2.8 nM and 0 nM. (Mean +/- SD, *n* = 3)

Here, human HK was incubated with living schistosomes (5 males) or commercially obtained human kallikrein (1 μg) and samples were recovered 6 h and 24 h later. Under these conditions, HK is cleaved by both schistosomes and kallikrein (as shown by western blot analysis, Fig. 2, described above). Samples were then diluted, added in replicate to a bradykinin ELISA plate to monitor bradykinin generation, and processed as described in methods. The 6 h and 24 h results are essentially the same and Fig. 4 shows the results of this analysis for the 24 h time point. It is clear that while, as expected, kallikrein generated easily detectable bradykinin (HK + Kallikrein), adult male schistosomes did not (HK + Males); no schistosome-cleaved HK sample yielded detectable bradykinin. The values obtained for the “schistosome + HK” samples were indistinguishable from those obtained using either HK alone or the zero-bradykinin control sample (ANOVA: *F*_(2,6)_ = 4.07, *P* = 0.14). The positive (+) and negative (-) controls are a bradykinin standard (2.8 nM) and no bradykinin, respectively.

## Discussion

In this study, we examined the impact of schistosomes on the murine plasma proteome, to gain insight into how the parasites interact with these host vascular components. It is clear that even in a short period (one hour) schistosomes can change the composition of plasma quite considerably. Using 2D-DIGE, we identified several protein spots that were markedly different in plasma that contained worms compared to plasma without worms. The biggest difference concerns the blood protein high molecular weight kininogen (HK). Four prominent spots contain this protein or fragments thereof. This paper focuses exclusively on the impact of schistosomes on HK. Other changes to the murine proteome will be published elsewhere.

Two forms of HK are found in greater abundance in control plasma (identified as spots 1 and 2) and two forms are found in greater abundance in plasma that contained worms (spots 3 and 4). The peptides identified following trypsin digestion of recovered spots 1 and 2 span all domains of the protein. In contrast, peptides identified following trypsin digestion of recovered spots 3 and 4 span HK domains 1–4; none are derived from domains 5 or 6. This suggests that protein spot 3 and 4 are both carboxyl truncated forms of full-length HK (spots 1 and 2).

To confirm that schistosomes were capable of cleaving HK (and not just in the context of murine plasma), worms were first incubated in buffer containing commercially obtained HK. Next, samples were recovered, resolved by SDS-PAGE, blotted to a membrane and probed with polyclonal anti-HK antibodies. This analysis confirms that the parasites are indeed capable of cleaving HK. Most prominent is the appearance of a 40 kDa fragment and, following prolonged incubation, the appearance of a 16 kDa fragment. Both intravascular larval stage parasites (schistosomula) and adult male worms are capable of cleaving HK to generate this characteristic digestion pattern. This pattern is distinct from that generated following cleavage of HK by the host enzyme kallikrein, which generates two characteristic major fragments (as well as the 9-amino acid peptide bradykinin).

While our finding is that parasite cleavage of HK is unlike that of host kallikrein cleavage of HK, it has been reported that *S. mansoni* homogenates do possess a kallikrein-like activity; this is associated with an uncharacterized 66 kDa protein, sK1, that has been described as hydrolyzing the kallikrein synthetic substrate d-Pro-Phe-Arg-p-nitroanilide [13]. Also, an *S. mansoni* cDNA encoding a ~35 kDa murine kallikrein homolog was identified, SmSP1 [14]. Localization of sK1 and SmSP1 suggest that they might be host-interactive and could, therefore, endow schistosomes with a kallikrein-like activity in vivo. Thus, in this work we set out to determine if intact, living schistosomes could be directly demonstrated to exhibit kallikrein activity by incubating live worms with the kallikrein substrate noted above. From this work, we find no evidence that live worms cleave this reagent. As a positive control, kallikrein, as expected, gives rise to rapid and sustained substrate cleavage and this activity can be inhibited by the addition of the serine protease inhibitor, PMSF. We, therefore, hypothesize that the substrate-cleaving sK1 schistosome protein is not host exposed and therefore plays no role in parasite interaction with HK.

As noted earlier, HK is the source of the 9-amino acid vasodilatory peptide bradykinin and kallikrein-mediated cleavage of HK releases bradykinin [12, 20]. We have shown above that schistosomes can cleave HK, yet they do not possess kallikrein activity. We are interested in learning whether the worms nonetheless retain the ability to generate bradykinin (in a non-kallikrein manner). We argue that being able to generate bradykinin might benefit the worms because of its vasodilatory properties; it could help the parasites control blood movement around them. Bradykinin can additionally act on the vascular endothelium to induce the production of the prostaglandin PGI_2_ [11]. PGI_2_ is an inhibitor of platelet activation, an activity that would also assist schistosomes. On the other hand, neutrophil, as well as mast cell, activation has been functionally linked to bradykinin production, which may be damaging for the parasites [21]. In this work, we directly measured bradykinin production following parasite-mediated cleavage of HK. No bradykinin was detected after parasite treatment. In contrast, kallikrein-mediated cleavage of HK yields easily measurable levels of bradykinin. This work confirms the observation that living schistosomes do not possess kallikrein activity; while the worms can cleave HK, they do not generate detectable levels of bradykinin. Whether further cleavage of the schistosome-generated HK fragments by host proteases leads to the eventual release of bradykinin is not known. One caveat arises from the report that an *S. mansoni* prolyl oligopeptidase (SmPOP) can degrade bradykinin [22]. This protein is found in the adult worm parenchyma and the tegument but no tegumental proteomic study finds it at the host-parasite interface [23–26]. Nonetheless, live adult worms are reported to cleave bradykinin suggesting that SmPOP on worms can access the peptide [22]. These data leave open the unlikely possibility that schistosomes can generate bradykinin, but it is rapidly broken down via SmPOP.

**Fig. 5:**
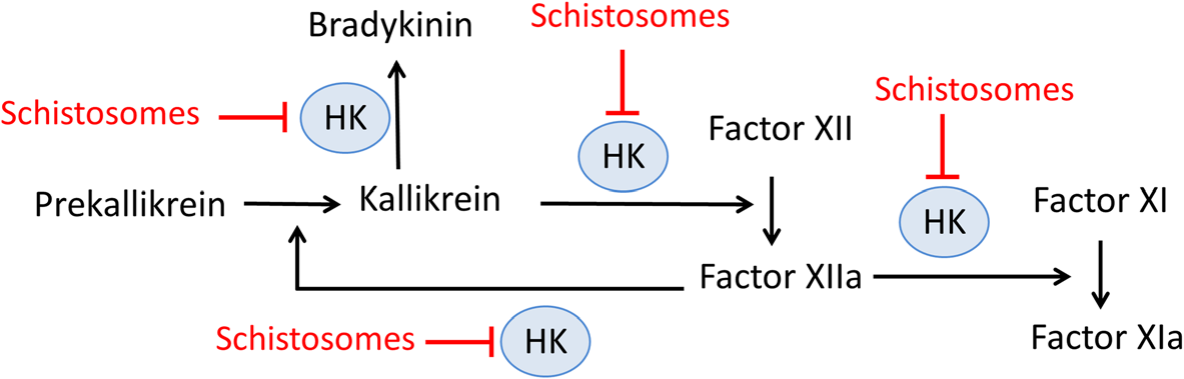
Depiction of biochemical pathways involving high molecular weight kininogen (HK, blue circles) that schistosomes may disrupt (red). HK is a cofactor in the conversion of coagulation factor XII to its active form (Factor XIIa) and the conversion factor XI to its active form (Factor XIa). HK is also involved in the Factor XIIa cleavage of prekallikrein to its active form, kallikrein. Finally, HK is cleaved by kallikrein to generate bradykinin. Cleavage of HK by parasites has the potential to disrupt all of these pathways

One reason that schistosomes cleave HK may be to disrupt its normal function in the initiation of blood coagulation. Figure 5 highlights the involvement of HK in several aspects of host metabolism. For example, HK acts as a co-factor in the conversion of factor XII to its active form (factor XIIa). Activated factor XIIa converts prekallikrein to kallikrein which then activates more factor XII. HK is an important cofactor in both reactions. HK is also necessary for the activation of factor XI by factor XIIa [11]. By cleaving a vital co-factor (HK) in the initiation of blood coagulation, the worms may impede blood clot formation around them in vivo. Certainly, worms within blood vessels do not seem perturbed by thromboses [20, 27] and ex vivo they can severely impede the ability of blood to clot [28]. However, whether HK cleavage by itself would necessarily impede coagulation remains to be determined; kallikrein cleavage of HK is reported to enhance its coagulant activity [29]. A lot depends on precisely where schistosomes cleave the HK molecule to destroy, or expose, specific domains. HK domain 3, in recombinant form, can inhibit thrombin-induced platelet aggregation [29]; domain 5 too has been identified as a potent inhibitor of platelet aggregation [29]. We hypothesize that generating fragments containing these domains may also benefit intravascular worms by further obstructing coagulation.

Impeding the activation of factor XII (by schistosome mediated HK cleavage) may have benefits for the worms beyond an impact on coagulation. For instance, since factor XIIa stimulates neutrophil aggregation [30], interleukin-1 expression in monocytes [31], and initiates the classical complement cascade [32], the ability of the worms to block its formation may additionally minimize the concentration of inflammatory mediators in their vicinity.

The question remains as to how schistosomes cleave HK to generate the characteristic pattern of protein fragments. Since the host HK cleaving enzyme kallikrein is a serine protease, we first set out to determine if the worms also possessed surface serine proteases that might be responsible for HK cleavage. To test this, parasites were incubated with HK plus PMSF (a serine protease inhibitor) but this did not impede the worm’s ability to cleave HK. In contrast, when the parasites are incubated with the cysteine protease inhibitor E64c, their HK cleaving ability was severely impaired. This result provides strong evidence for the view that schistosome cysteine proteases are responsible for HK cleavage. This result is also consistent with tegumental proteomic work which places cysteine proteases belonging to the calpain family in the parasite surface membranes but no serine proteases there [24, 25]. Calpains are calcium-dependent cysteine proteases that engage in limited cleavage of their target proteins [33–35].

We have cloned the two calpain proteases identified by proteomic analysis as being expressed in the *S. mansoni* tegument. These are designated SmCalp1 and SmCalp2 and they display a highly conserved classical calpain domain organization [36]. Both are highly expressed in the parasite’s intravascular life forms. Immunolocalization and activity-based protein profiling experiments confirm their presence at the host-parasite interface [36]. Furthermore, we have shown that living parasites exhibit surface calpain activity [36]. While we show here that live worms do not cleave a kallikrein substrate (D-Pro-Phe-Arg-pNa), earlier work shows that living worms can cleave a calpain substrate (DABCYL-Thr-Pro-Leu-Lys-Ser-Pro-Pro-Pro-Ser-Pro-Arg-EDANS) [36]. Cleavage of the latter is blocked in the absence of calcium and the presence of the calpain inhibitor E64c [36]. While calpains are invariably reported to be exclusively intracellular (except in diseased or injured tissues), it appears that schistosomes display unique, constitutive, functional extracellular calpain activity. In this work, we show that schistosomes can cleave the blood clotting protein HK in a manner that is inhibitable by the non-cell permeable, calpain inhibitor E64c. Since domains 2 and 3 of HK are designated as being involved in “cysteine protease inhibition” [37, 38], it is ironic that schistosome cysteine proteases may cleave this protein.

Other pathogens have been shown to interact with HK (e.g. the fungus *Candida* spp. [39, 40]) and some possess proteases that cleave HK (e.g. the bacteria *Streptococcus pyogenes* [41] and *Porphyromonas gingivalis* [42]). Our work is the first to show that a metazoan parasite (*S. mansoni*) can also cleave HK and our data differ from that reported for the bacterial pathogens in that no bradykinin is generated following worm-mediated HK cleavage.

In previous work, we showed that schistosomes are also capable of cleaving another host blood clotting protein, fibronectin, and that this activity can also be inhibited by E64c [36]. We hypothesize that the cysteine proteases SmCalp1 and/or SmCalp2 cleave both fibronectin and HK and towards the same end, to help impede blood clot formation around the worms in vivo and to diminish the concentration of inflammatory mediators near them. Schistosomes possess other molecular mechanisms that have been hypothesized to help prevent local thromboses [20]. These include their possession of ectoenzymes that can cleave pro-thrombotic nucleotides like ADP [8] as well as surface molecules that can promote the generation of active plasmin, a major clot-degrading enzyme [9]. These combined capabilities would promote the ease of unrestricted movement of the parasites within the vasculature of their hosts.

## Conclusions

We have shown in this study that even with a short period of time schistosomes can clearly have an impact on the murine plasma proteome, specifically on high molecular weight kininogen. With our previous work on identification of two cysteine proteases belonging to the calpain family (SmCalp1 and SmCalp2) on the surface of schistosomes, we show and conclude that they are likely responsible for the HK cleavage reported here. The cleavage of HK is probably another factor how schistosomes impede blood clotting and inflammation around them in vivo and so promote their ease of movement within the vasculature of their hosts.

## Additional file

Additional file 1: **Table S1.** Sequences of peptides identified by mass spectrometry from protein spots 1–4 (as shown in Fig. 1a). Spots 1 and 2 are from plasma sample obtained in the absence of parasites (green) and spots 3 and 4 are from plasma sample that contained adult schistosomes (red), as described in methods. An “x” indicates that the peptide was found in the indicated protein spot. The amino acid start and end positions of each peptide within the murine high molecular weight kininogen (accession designation: KNG1_MOUSE) sequence are given. (PDF 418 kb)

## Abbreviations

HK: High molecular weight kininogen
NIH-NIAID: National Institutes of Health-National Institute of Allergy and Infectious Diseases
BRI: Biomedical Research Institute
SmSP1: *Schistosoma mansoni* serine protease 1
DMEM/F12: Dulbecco's Modified Eagle Medium/Nutrient Mixture F-12
IACUC: Institutional Animal Care and Use Committees
2D-DIGE: Two-dimensional differential in-gel electrophoresis
SDS-PAGE: Sodium dodecyl sulfate polyacrylamide gel electrophoresis
MS: Mass spectrometry
HBSS: Hanks balanced salt solution
PMSF: Phenylmethylsulfonyl fluoride
TMB: Tetramethylbenzidine
SmPOP: *Schistosome mansoni* prolyl oligopeptidase
