# Supplementary material for: The blood fluke Schistosoma mansoni cleaves the coagulation protein high molecular weight kininogen (HK) but does not generate the vasodilator bradykinin
Source: Parasit Vectors. 2018 Mar 14;11:182. doi: 10.1186/s13071-018-2704-0 (PMC5853081; doi:10.1186/s13071-018-2704-0)
Supplement: Supplementary file 1 — Table S1. Sequences of peptides identified by mass spectrometry from protein spots 1–4 (as shown in Fig. 1a). Spots 1 and 2 are from plasma sample obtained in the absence of parasites (green) and spots 3 and 4 are from plasma sample that contained adult schistosomes (red), as described in methods. An “x” indicates that the peptide was found in the indicated protein spot. The amino acid start and end positions of each peptide within the murine high molecular weight kininogen (accession designation: KNG1_MOUSE) sequence are given. (PDF 418 kb) [file 13071_2018_2704_MOESM1_ESM.pdf]

Table S1

Sequences of peptides identified by mass spectrometry from protein spots 1 – 4 (as shown in figure 1A). Spots 1 and 2 are from plasma sample obtained in the absence of parasites (green) and spots 3 and 4 are from plasma sample that contained adult schistosomes (red), as described in methods. An "x" indicates that the peptide was found in the indicated protein spot. The amino acid start and end positions of each peptide within the murine high molecular weight kininogen (accession designation: KNG1_MOUSE) sequence are given.

Start	End	Sequence	Spot 1	Spot 2	Spot 3	Spot 4
50	58	SGNQYMLHR	X	X	X	X
65	75	TDGSPTFYSFK	X	X	X	X
98	113	DAEEAATGECTATVGK			X	X
114	126	RENEFFIVTQTCK		X		
115	126	ENEFFIVTQTCK	X	X	X	X
115	131	ENEFFIVTQTCKIAPSK			X	X
137	160	AYFPCIGCVHAISTDSPDLEPVLK	X	X		X
208	229	ERFPSLHGDCVALPNGDDGECR	X	X	X	X
210	229	FPSLHGDCVALPNGDDGECR	X	X	X	X
230	239	GNLFMDINNK	X	X	X	X
269	278	DIPVDSPELK				X
279	299	EVLGHSIAQLNAENDHPFYYK		X	X	X
306	315	ATSQVVAGTK				X
316	323	YVIEFIAR	X	X	X	X
330	342	ESNTELAEDCEIK	X	X	X	X
343	362	HLGQSLDCNANVYMRPWENK	X	X	X	X
369	379	CQALDMTEMAR		X		
369	388	CQALDMTEMARRPPGFSPFR			X	
380	388	RPPGFSPFR	X	X		
400	408	TVSPPYIAR			X	X
409	431	EQEERDAETEQGPTHGHGWLHEK	X			
414	431	DAETEQGPTHGHGWLHEK	X			
444	454	HGHDHGHWSPR	X			
455	475	RHGLGHGHQKPHGLGHGHQLK	X			
456	475	HGLGHGHQKPHGLGHGHQLK	X			
619	630	LISDFPEATSPK	X	X		
